# Phase II study of docetaxel in patients with metastatic pancreatic cancer: a Japanese cooperative study

**DOI:** 10.1038/sj.bjc.6690375

**Published:** 1999-05-01

**Authors:** S Okada, Y Sakata, S Matsuno, M Kurihara, Y Sasaki, Y Ohashi, T Taguchi, for the Cooperative Group of Docetaxel for Pancreatic Cancer in Japan

**Affiliations:** Department of Internal Medicine, National Cancer Center Hospital, 5-1-1 Tsukiji, Chuo-ku, Tokyo, 104-0045, Japan; Department of Gastroenterology and Endoscopy, Aomori Prefectural Central Hospital, 2-1-1 Higashi Tsukurimichi, Aomori-shi, Aomori, 030-0913, Japan; Department of Surgery I, Tohoku University School of Medicine, 2-1 Seiryocho, Aoba-ku, Sendai-shi, Miyagi, 980-0873, Japan; Department of Gastroenterology, Toyosu Hospital, Showa University, 4-1-18 Toyosu, Koto-ku, Tokyo, 135-8577, Japan; Department of Oncology/Hematology and Clinical Pharmacology, National Cancer Hospital East, 6-5-1 Kashiwanoha, Kashiwa-sh, Chiba, 277-0882, Japan; Department of Epidemiology and Biostatistics, School of Health Science and Nursing, Faculty of Medicine, University of Tokyo, 7-3-1 Hongo, Bunkyo-ku, Tokyo, 113-0033, Japan; Japan Society for Cancer Chemotherapy, 1-18-35 Edobori, Osaka, 550-0014, Japan

**Keywords:** docetaxel, chemotherapy, pancreatic cancer, phase II study

## Abstract

Docetaxel has been reported to show promising anti-tumour activity in pancreatic ductal cancer (PC). This study was conducted to evaluate the activity and toxicity of moderate-dose (60 mg m^−2^) docetaxel in Japanese chemo-naive patients with measurable metastatic PC. The patients had a performance status of 0–2. They received docetaxel intravenously over a 1- to 2-h period without any premedication for hypersensitivity reactions. This treatment was repeated every 3–4 weeks with dose adjustments based on the toxic effects observed. Twenty-one patients were eligible and treated with docetaxel. The median number of courses was 2 (range, 1–4). None of the patients achieved an objective response; seven showed no change and 13 showed progressive disease. In one patient, the response was not assessable because of early death. The median survival time for all patients was 118 days. The main grade 3–4 toxicities by patient were leucocytopenia (67%) and neutropenia (86%). Other grade 3–4 toxicities included anaemia (10%), thrombocytopenia (5%), nausea/vomiting (29%), anorexia (29%), GOT/GPT increase (10%), alkaline phosphatase increase (14%), malaise/fatigue (33%) and alopecia (24%). In conclusion, docetaxel, administered on this schedule, did not show significant anti-tumour activity in patients with metastatic PC. © 1999 Cancer Research Campaign


*British Journal of Cancer* (1999) **80**(3/4), 438–443
© 1999 Cancer Research Campaign
Article no. bjoc.1998.0375

### Phase II study of docetaxel in patients with metastatic pancreatic cancer: a Japanese cooperative study

**S Okada[1], Y Sakata[2], S Matsuno[3], M Kurihara[4], Y Sasaki[5], Y Ohashi[6] and T Taguchi[7], for the Cooperative Group of Docetaxel for Pancreatic Cancer in Japan**

[1]Department of Internal Medicine, National Cancer Center Hospital, 5-1-1 Tsukiji, Chuo-ku, Tokyo, 104-0045, Japan; [2]Department of Gastroenterology and Endoscopy, Aomori Prefectural Central Hospital, 2-1-1 Higashi Tsukurimichi, Aomori-shi, Aomori, 030-0913, Japan; [3]Department of Surgery I, Tohoku University School of Medicine, 2-1 Seiryocho, Aoba-ku, Sendai-shi, Miyagi, 980-0873, Japan; [4]Department of Gastroenterology, Toyosu Hospital, Showa University, 4-1-18 Toyosu, Koto-ku, Tokyo, 135-8577, Japan; [5]Department of Oncology/Hematology and Clinical Pharmacology, National Cancer Hospital East, 6-5-1 Kashiwanoha, Kashiwa-shi, Chiba, 277-0882, Japan; [6]Department of Epidemiology and Biostatistics, School of Health Science and Nursing, Faculty of Medicine, University of Tokyo, 7-3-1 Hongo, Bunkyo-ku, Tokyo, 113-0033, Japan; [7]Japan Society for Cancer Chemotherapy, 1-18-35 Edobori, Nishi-ku, Osaka, 550-0014, Japan

**Summary** Docetaxel has been reported to show promising anti-tumour activity in pancreatic ductal cancer (PC). This study was conducted to evaluate the activity and toxicity of moderate-dose (60 mg $m^{-2}$) docetaxel in Japanese chemo-naive patients with measurable metastatic PC. The patients had a performance status of 0–2. They received docetaxel intravenously over a 1- to 2-h period without any premedication for hypersensitivity reactions. This treatment was repeated every 3–4 weeks with dose adjustments based on the toxic effects observed. Twenty-one patients were eligible and treated with docetaxel. The median number of courses was 2 (range, 1–4). None of the patients achieved an objective response; seven showed no change and 13 showed progressive disease. In one patient, the response was not assessable because of early death. The median survival time for all patients was 118 days. The main grade 3–4 toxicities by patient were leucocytopenia (67%) and neutropenia (86%). Other grade 3–4 toxicities included anaemia (10%), thrombocytopenia (5%), nausea/vomiting (29%), anorexia (29%), GOT/GPT increase (10%), alkaline phosphatase increase (14%), malaise/fatigue (33%) and alopecia (24%). In conclusion, docetaxel, administered on this schedule, did not show significant anti-tumour activity in patients with metastatic PC.

**Keywords**: docetaxel; chemotherapy; pancreatic cancer; phase II study

Pancreatic ductal cancer (PC) is difficult to treat, with most patients surgically unresectable at the time of diagnosis. Moreover, even for those who are resected, the risk of recurrence is exceedingly high, and the outcome remains unsatisfactory. The prognosis of unresectable PC patients is also extremely poor, mainly because currently available chemotherapeutic agents are largely ineffective. Accordingly, there is a clear need for new, effective agents in the management of PC. New agents with unique mechanisms of action are attractive candidates for clinical trials with the hope that their anti-tumour activity will be translated into long-term survival.

Docetaxel, which is a member of the family of taxanes, is one of the most important new chemotherapeutic agents to be found in recent years (Bissett et al, 1993; Cortes and Pazdur, 1995; Eisenhauer, 1995). It acts by enhancing microtubule assembly and inhibiting tubulin depolymerization, thus disrupting cell division (Schiff et al, 1979; Guéritte-Voegelein et al, 1991; Rowinsky and Donehower, 1995). This mechanism of action is unlike any of the standard cytotoxic agents, and therefore docetaxel has the potential for activity against human solid tumours, including PC, that are refractory to established anti-cancer agents. In fact, two phase II trials of advanced PC, which were conducted in France and the USA, have reported high response rates (20%) and relatively longer survival with the drug administered at 100 mg $m^{-2}$ over a 1-h period (Rougie et al, 1994; Abbruzzese et al, 1995).

We report here our results of a cooperative phase II study of moderate-dose (60 mg $m^{-2}$) docetaxel in Japanese patients with previously untreated metastatic PC. In our country, the recommended dose for phase II trials of docetaxel is 60 mg $m^{-2}$ infused over a 1-h session, because a Japanese phase I trial determined the maximum-tolerated dose (MTD) to be 70–90 mg $m^{-2}$, with leucocytopenia as the dose-limiting toxicity (Taguchi et al., 1994).

*Received 8 July 1998*
*Revised 13 November 1998*
*Accepted 20 November 1998*

*Correspondence to:* S Okada

### PATIENTS AND METHODS

### Patients

Patients eligible for study entry had metastatic PC for which they had not received any treatment. Each patient was required to meet the following eligibility criteria: a performance status (PS) of 0–2; 15–74 years of age; at least one bidimensionally measurable tumour; estimated life expectancy ≥ 2 months after study entry; adequate renal function (normal serum creatinine and blood urea nitrogen levels); adequate liver function (total bilirubin level ≤ 1.5 mg $dl^{-1}$ (or ≤ 3.0 mg $dl^{-1}$ after biliary drainage if the patient had obstructive jaundice); adequate serum transaminases (GOT, GPT) levels ≤ 2 times upper normal limit (UNL) (or ≤ 3 times

UNL in the patients with liver metastases and/or obstructive jaundice) and serum alkaline phosphatase ≤ 1.5 times UNL (or ≤ 2.5 times UNL in the patients with liver metastases and/or obstructive jaundice); adequate bone marrow reserve (white blood cell count ≥ 4000 $mm^3$ and ≤ 10 000 $mm^3$, neutrophil count ≥ 2000 $mm^3$, platelet count ≥ 100 000 $mm^3$, and haemoglobin level ≥ 9.5 g $dl^{-1}$); and written informed consent.

The exclusion criteria were as follows: active infection; severe heart disease; interstitial pneumonitis or pulmonary fibrosis; ≥ grade 2 peripheral neuropathy by the Japan Society for Cancer Therapy (JSCT) toxicity criteria (Japan Society for Cancer Therapy, 1993); known metastases of the central nervous system; active concomitant malignancy; pregnant and lactating females; females of childbearing age unless using effective contraception; concurrent treatment with corticosteroids; history of drug-hypersensitivity; pleural or pericardial effusion that required drainage; peripheral oedema; other serious medical conditions.

The number of patients to be enrolled was planned using a modified multi-stage Fleming design based on the assumptions that expected response rate of docetaxel was 15%, response rate to be judged no activity was 5%, α error was 5% (one-tailed) and β error was 10% (Fleming, 1982; Simon, 1989). In case of no response, the number of 19 patients provides that the lower limit of 95% confidence interval (CI) is ≤ 15%. However, interim analysis was planned to be done when 20–25 patients were enrolled, because some eligible patients often had not been evaluable for the activity in clinical trials of metastatic PC. If none of the first 20–25 patients had a partial or complete response, the trial was to be ended. If a major objective response was detected in any of the first 20–25 patients studied, an additional 20–25 patients were to be studied in a second stage of accrual to estimate more precisely the actual response rate.

### Treatments

Patients were admitted to hospitals during the chemotherapy courses. Docetaxel was supplied by Rhone-Poulenc Rorer Pharmaceuticals Inc., Antony, France, in a concentrated sterile vial that contained 80 mg of the drug in 2 ml of polysorbate 80. The starting dose of 60 mg $m^{-2}$ was diluted in 250–500 ml of 5% glucose or 0.9% saline, and was infused over a 1- to 2-h period. No routine premedication for hypersensitivity reactions was given, and there was no routine prophylactic administration of anti-emetics or granulocyte colony-stimulating factors. The treatment was repeated every 3–4 weeks, provided the patient had sufficiently recovered from toxicity, and was continued until there was evidence of disease progression or unacceptable toxicity.

The dose of docetaxel was adjusted according to haematological and other toxicities observed. Patients without ≥ grade 3 toxicity during the first course could receive 70 mg $m^{-2}$ in subsequent courses. Patients who experienced grade 4 leucocytopenia or neutropenia that lasted ≥ 5 days, or had a treatment delay of > 2 weeks due to incomplete recovery from toxicity, received 50 mg $m^{-2}$ in subsequent courses. The treatment was stopped when ≥ grade 3 toxicities other than haematological, nausea/vomiting, anorexia, malaise/fatigue, or alopecia occurred.

When hypersensitivity reactions occurred, the docetaxel administration was stopped, and corticosteroids and antihistamines were given. Patients who experienced hypersensitivity reactions were pretreated with these drugs in subsequent courses. Patients who experienced ≥ grade 2 nausea/vomiting were pretreated with anti-emetics in subsequent courses. With respect to leucocytopenia or neutropenia, lenograstim (Neutrogin; Chugai Pharmaceuticals Inc., Tokyo, Japan) was administered subcutaneously when grade 4 toxicity or grade 3 toxicity with fever occurred.

### Response and toxicity evaluation

The primary end point of this study was to evaluate the activity and toxicity of docetaxel in metastatic PC. The anti-tumour response was assessed at least every 3–4 weeks. In this study, sizes of metastatic lesions were measured to evaluate objective tumour response to docetaxel; however, pancreatic masses were not considered to be measurable but only assessable, because imaging modalities including ultrasonography (US) and computerized tomography (CT) may be insufficient to accurately determine the tumour size of pancreatic masses. The best overall response (best response category achieved between the start of docetaxel treatment and the onset of progression) was recorded for each patient. The duration of response (dated from study entry), time to progression (dated from study entry), and duration of survival (dated from study entry) were also calculated by the Kaplan–Meier method. All responses were strictly judged by extramural review.

The clinical response to docetaxel treatment was evaluated by PS and pain. PS was recorded weekly by physicians, and patients with a PS of 1 or 2 were defined as eligible, because a patient with a PS of 0 did not have potential to improve. Pain was evaluated by measuring changes from the baseline in pain intensity and morphine consumption. Patients who met at least one of the following criteria were defined as eligible for the evaluation of pain: (1) baseline pain intensity of ≥ 20 (out of 100) as measured by the pain assessment card, and (2) baseline morphine consumption of ≥ 10 mg $day^{-1}$. Each of the eligible patients recorded pain intensity on a pain assessment card every 2 weeks.

We used the JSCT criteria, which are fundamentally similar to the World Health Organization (WHO) criteria (Miller et al, 1981), for evaluating the responses and the toxic effects. A monitoring committee was arranged independently to assess the evaluation of efficacy and safety in the study.

This trial was designed in accordance with Japanese guidelines for the clinical evaluation of anti-neoplastic drugs (The Ministry of Health and Welfare, 1991), and was performed after the approval of the investigational review board of each hospital was given.

### RESULTS

### Patients

Twenty-two patients were enrolled in the study from 12 hospitals across Japan between October 1995 and December 1996 (Registration Office, Japanese Society for Cancer Chemotherapy). One patient was retrospectively found to be ineligible (he had acinar cell carcinoma rather than adenocarcinoma) and was excluded from the analysis. Thus, 21 metastatic PC patients were eligible and evaluable for both the activity and toxicity. The diagnosis of PC was confirmed by histological examination in 15 patients and, in the remaining six patients, it was based on typical radiographic findings of PC. However, in three of these six patients, the diagnosis of PC was confirmed histologically by autopsy.

The baseline characteristics of the 21 eligible patients are summarized in Table 1. There were 11 males and ten females with

© Cancer Research Campaign 1999

**Table 1** Profile of the metastatic pancreatic cancer patient population

| Characteristics | No. | % |
|---|---|---|
| No. of patients | 21 | |
| Gender | | |
| Male | 11 | 52 |
| Female | 10 | 48 |
| Age, years | | |
| Median | 58 | |
| Range | 43–70 | |
| ECOG[a] performance status | | |
| 0 | 10 | 48 |
| 1 | 7 | 33 |
| 2 | 4 | 19 |
| Prior therapy | | |
| Pancreatectomy | 1 | 5 |
| Palliative surgery | 3 | 14 |
| Radiotherapy | 2 | 10 |
| Liver dysfunction | 15 | 71 |
| Sites of metastases | | |
| Liver | 17 | 81 |
| Lymph nodes | 8 | 38 |
| Lung | 1 | 5 |
| Ovary | 1 | 5 |
| No. of metastatic sites | | |
| 1 | 15 | 71 |
| 2 | 6 | 29 |
| No. of treatment courses | | |
| 1 | 7 | 33 |
| 2 | 7 | 33 |
| 3 | 4 | 19 |
| 4 | 3 | 14 |

[a]ECOG: Eastern Cooperative Oncology Group a median age of 58 years (range 43–70). Eleven patients (52%) had symptomatic disease (PS 1 or 2). One patient had undergone a pancreatectomy with intraoperative radiotherapy, and another patient had received previous radiation therapy to the pancreatic mass. Before chemotherapy, three patients underwent biliary drainage for obstructive jaundice. Six patients (29%) showed normal liver function, and the remaining 15 (71%) had liver dysfunction. Seventeen patients (81%) had liver metastasis. Among them, 16 had liver involvement of less than 25% of the entire liver, and the remaining one patient had involvement of 25–50%. Fifteen patients (71%) had one metastatic site and six (29%) had two metastatic sites.

### Treatments

The 21 patients eligible for the study were given a total of 45 courses, with a median of two courses each (range 1–4). The dose of docetaxel was modified for six patients; dose reduction to 50 mg $m^{-2}$ was necessary in two of the three courses as a result of myelosuppression, and dose escalation to 70 mg $m^{-2}$ was done in four of the eight courses because of good tolerability during the prior course. The median cumulative dose of docetaxel received was 120 mg $m^{-2}$, with a range of 60–270 mg $m^{-2}$. The reasons for treatment discontinuation were: disease progression (18 patients; 86%), patient's refusal of treatment (one patient; 5%) and toxicity (two patients – one early death due to neutropenic sepsis and one neurosensory disorder; 9%). After docetaxel treatment, six patients received systemic chemotherapy, one radiotherapy for pancreatic mass and one chemoradiation for pancreatic mass. However, no patient achieved an objective tumour response. The other 13 patients underwent only the best supportive care after docetaxel treatment.

During the study, five patients may have received about 10% overdose due to an inadequate preparation of docetaxel. Therefore, for the statistical analysis, two patient populations were defined: the intent-to-treat population which included all eligible patients (21 patients); and the population which included the eligible patients who received docetaxel at the scheduled dose (16 patients).

### Response

Although the response could not be determined in one patient due to an early death on day 8 of the first course, no objective response was observed, giving an overall response rate of 0% (95% CI, 0–15%). Seven (33%) of the eligible patients showed no change (NC) with a median duration from study entry of 98 days (range 50–168 days). Among these NC patients, one patient achieved a 61% decrease in the size of liver metastasis, but this condition did not last for 4 weeks. The remaining 13 (62%) patients showed progressive disease (PD). The patients who achieved NC received a median of three courses (range 2–4), and those with PD received a median of two courses (range 1–3).

Progression of disease was seen in 20 (95%) of the 21 patients, and the remaining one patient was not evaluable for time to progression because of an early death. The median time to progression was 36 days (range 14–168 days). All but two patients died, with the median survival time of 118 days (95% CI, 105–158 days). Two patients had survived for more than 1 year (607+ and 596+ days) at the time of analysis. Among the 16 patients who received docetaxel at the scheduled dose, the median time to progression was 41 days (range 16–168 days), with the median survival time of 109 days (95% CI, 99–131 days).

With regard to clinical response, one (9%) of the 11 eligible patients with a PS of 1 or 2 experienced an improvement in PS. The responder had a PS of 1 before chemotherapy, and his general condition improved after three courses of docetaxel, i.e. a PS of 0 sustained for 6 weeks. Among the 13 eligible patients in the evaluation of pain, ten had an increase from baseline and the remaining three showed no remarkable change in morphine consumption. One (8%) of the three patients with no increase in morphine consumption achieved an improvement of ≥ 50% from baseline in pain intensity for 8 weeks.

### Toxicity

The haematological toxicity of docetaxel is summarized in Table 2. All but one patient were assessed for toxicity. One patient died of neutropenic sepsis on day 8 of the first course, before the evaluation of toxicity was complete. The docetaxel therapy was generally well-tolerated, with haematological toxicity, most notably reversible neutropenia, being the most common severe toxicity of docetaxel on this schedule. Grade 3–4 leucocytopenia and neutropenia occurred in 14 (67%) and 18 (86%) of the patients respectively. Grade 4 leucocytopenia occurred in two (10%) patients and two (4%) courses, and neutropenia occurred in 11 (52%) patients and 16 (36%) courses. However, the leucocytopenia and neutropenia were brief and reversible, although these patients received lenograstim; the median time to the nadir of the neutropenia was 8 days, and the median time from the nadir to

 © Cancer Research Campaign 1999

**Table 2** Toxicity of docetaxel

| | Grade | | | |
|---|---|---|---|---|
| **Toxicity** | **1** | **2** | **3** | **4** |
| Haematological toxicity | | | | |
| Per patient | | | | |
| Anaemia | 8 | 3 | 2 | 0 |
| Leucocytopenia | 1 | 5 | 12 | 2 |
| Neutropenia[a] | 0 | 2 | 7 | 11 |
| Thrombocytopenia | 3 | 1 | 1 | 0 |
| Per course | | | | |
| Anaemia | 14 | 4 | 2 | 0 |
| Leucocytopenia | 6 | 10 | 20 | 2 |
| Neutropenia | 3 | 3 | 17 | 16 |
| Thrombocytopenia | 3 | 1 | 1 | 0 |
| Non-haematological toxicity (per patient) | | | | |
| Gastrointestinal | | | | |
| Bilirubin | 2 | 1 | 0 | 0 |
| GOT | 2 | 4 | 1 | 1 |
| GPT | 1 | 5 | 2 | 0 |
| ALP[b] | 0 | 3 | 3 | 0 |
| Anorexia | 3 | 6 | 6 | 0 |
| Nausea/vomiting | 5 | 4 | 6 | 0 |
| Diarrhoea | 4 | 3 | 0 | 0 |
| Fever | 4 | 10 | 0 | 0 |
| Skin rash | 6 | 3 | 0 | 0 |
| Alopecia | 4 | 9 | 5 | 0 |
| Sensory neuropathy | 0 | 1 | 1 | 0 |
| Malaise/fatigue | 6 | 2 | 5 | 2 |
| Others[c] | 4 | 4 | 1 | 0 |

[a]One death due to neutropenic sepsis. [b]ALP: alkaline phosphatase. [c]Includes proteinuria ($n$ = 1, grade 3), back pain ($n$ = 1, grade 2), hyperventilation ($n$ = 1, grade 2), facial flush ($n$ = 1, grade 2), nail change ($n$ = 1, grade 2), pruritus ($n$ = 1, grade 1), electrocardiography abnormality ($n$ = 1, grade 1), headache ($n$ = 1, grade 1) and peripheral oedema ($n$ = 1, grade 1).

recovery (≥ 2000 ml) was 8 days. Febrile episodes occurred in 14 (67%) patients and a neutropenic fever, defined as fever (> 38°C) concomitant with grade 4 neutropenia, occurred in six (29%) patients and six (13%) courses. Thrombocytopenia and anaemia were infrequent and mild. No cumulative tendency of myelosuppression was noted as the treatment courses continued.

The non-haematological toxicities of docetaxel in this study, summarized in Table 2, were generally mild and well-tolerated. Nausea/vomiting, anorexia, malaise/fatigue and alopecia were the most common non-haematological toxicities. Although the majority of these events were mild, it was ≥ grade 3 in about one-third of the patients. Other grade 3 toxicities consisted of liver function abnormalities, sensory neuropathy and proteinuria. Diarrhoea was fairly common, as was skin rash, but these were both mild and transient. Despite having received no prophylactic premedication, no patient experienced hypersensitivity reactions, and only one patient experienced grade 1 peripheral oedema.

With regard to the relationship between liver function and toxicities, the patients with liver dysfunction did not experience severe toxicities (≥ grade 3) more frequently compared to the patients with normal liver function, although dose reduction was needed in the two patients with liver dysfunction as a result of myelosuppression (Table 3). Toxicities in the 16 patients who received docetaxel at the scheduled dose mirrored the findings in all 21 eligible patients.

**Table 3** Toxicity of docetaxel with reference to liver function (per patient)

| | Liver function | |
|---|---|---|
| | **Normal ($n$ = 6)** | **Abnormal ($n$ = 15)** |
| | **Grades 3–4 (%)** | **Grades 3–4 (%)** |
| Haematological toxicity | | |
| Anaemia | 1 (17) | 1 (7) |
| Leucocytopenia | 6 (100) | 8 (53) |
| Neutropenia | 6 (100)[a] | 12 (80) |
| Thrombocytopenia | 1 (17) | 0 (0) |
| Non-haematological toxicity | | |
| Gastrointestinal | | |
| Bilirubin | 0 (0) | 0 (0) |
| GOT | 1 (17) | 1 (7) |
| GPT | 1 (17) | 1 (7) |
| ALP[b] | 2 (33) | 1 (7) |
| Anorexia | 2 (33) | 4 (27) |
| Nausea/vomiting | 2 (33) | 4 (27) |
| Diarrhoea | 0 (0) | 0 (0) |
| Fever | 0 (0) | 0 (0) |
| Skin rash | 0 (0) | 0 (0) |
| Alopecia | 2 (33) | 3 (20) |
| Sensory neuropathy | 0 (0) | 1 (7) |
| Malaise/fatigue | 3 (50) | 4 (27) |
| Others | 1 (17) | 0 (0) |

[a] One death due to neutropenic sepsis. [b]ALP: alkaline phosphatase.

### DISCUSSION

Clinical trials to date have demonstrated that docetaxel has significant and consistent anti-tumour activities in a variety of solid tumours, including breast cancer, non-small-cell lung cancer and ovarian cancer (Cerny et al, 1994; Francis et al, 1994; Adachi et al, 1996; Dieras et al, 1996; Kunitoh et al, 1996). With regard to PC, preclinical studies have shown docetaxel to be active in PC models (Bissery et al, 1991), and two clinical trials of docetaxel at 100 mg $m^{-2}$ have demonstrated a promising anti-tumour activity in PC (Rougie et al, 1994; Abbruzzese et al, 1995). In a French trial, partial responses were achieved in six (20%) of 30 patients with measurable liver metastases, with the median survival time of 212 days (Rougie et al, 1994). A trial reported from the USA showed preliminary results of two partial responses (20%) in ten assessable patients, with both responders showing improvement in cancer-related pain and PS (Abbruzzese et al, 1995). These results argued for the further evaluation of docetaxel in chemo-naive patients with metastatic PC to define the true usefulness of this agent.

The present study, in which 21 chemo-naive patients with metastatic PC were treated with moderate-dose (60 mg $m^{-2}$) docetaxel, failed to demonstrate an objective response against PC. Although a significant number of patients with NC were noted after docetaxel therapy, the duration of NC was brief, lasting a few months in the majority of the patients. The median survival time of 118 days, which was comparable to most phase II trials of metastatic PC, was much poorer than that in the French trail. Furthermore, the clinical response such as improvement in PS and/or pain was achieved in only a small portion of the patients. With regard to toxicity, the severity of neutropenia, the dose-limiting toxicity of this agent, was comparable between the current study and earlier trials with 100 mg $m^{-2}$ (Cortes and Pazdur,

© Cancer Research Campaign 1999

1995). In fact, in our study, nearly 80% of the patients experienced grade 3–4 neutropenia, and a considerable proportion of patients developed a neutropenic fever. In contrast to haematological toxicity, the non-haematological toxicity in this study was relatively different from that in earlier trials using a higher dosage (Cortes and Pazdur, 1995). Despite the fact that no routine premeditation was used, we observed no acute hypersensitivity reaction, and peripheral oedema was seen infrequently. The differences might be attributable to the differences in the dose per course and the cumulative dosage.

The results of the present study, especially the anti-tumour activities of docetaxel, are not consistent with those of the previous phase II trials that used the higher dose (100 mg $m^{-2}$) of docetaxel in advanced PC patients (Routine et al, 1994; Abbruzzese et al, 1995). The significantly lower dosage of the drug used here is the most likely explanation for this inconsistency. A higher dose may be clinically more effective, since the anti-tumour activity of docetaxel probably depends on the dose (Fumoleau et al, 1993; Adachi et al, 1996; Dieras et al, 1996). However, higher doses of docetaxel could not be used given the results of phase I trails in Japan (Taguchi et al, 1994). As described previously, a Japanese phase I trial determined the MTD to be 70–90 mg $m^{-2}$ and the recommended dose to be 60 mg $m^{-2}$ for further clinical trials. The reason for the lower MTD in Japanese patients remains to be elucidated. No racial difference was found in the elimination of docetaxel (Bruno et al, 1995; Tanigawara et al, 1996).

In conclusion, docetaxel, at a dose of 60 mg $m^{-2}$ as a 1- to 2-h intravenous infusion every 3–4 weeks, had no significant anti-tumour activity, with considerable haematological toxicities in chemo-naive metastatic PC patients. These data do not support the practical use of docetaxel in Japanese patients with this disease.

### ACKNOWLEDGEMENTS

This study was sponsored by Rhône-Poulenc Rorer Japan, Inc. and Chugai Pharmaceutical Co. Ltd. We wish to thank Ms Keiko Kondo for help with manuscript preparation.

### Appendix 1

Phase II Study of Docetaxel in Pancreatic Cancer Group

| | |
|---|---|
| Y Sakata | Department of Gastroenterology and Endoscopy, Aomori Prefectural Central Hospital |
| Y Sasaki | Department of Internal Medicine, Ishinomaki Red Cross Hospital |
| H Saito | Department of Internal Medicine, Yamagata Prefectural Central Hospital |
| H Saisho | Department of Internal Medicine I, School of Medicine, Chiba University |
| J Furuse | Department of Internal Medicine, National Cancer Center Hospital East |
| S Okada | Department of Internal Medicine, National Cancer Center Hospital |
| M Kurihara | Department of Gastroenterology, Toyosu Hospital, Showa University |
| J Ariyama | Department of Gastroenterology, Juntendo University School of Medicine |
| S Tamai | Department of Internal Medicine II, Kanagawa Cancer Center |
| K Ogoshi | Department of Internal Medicine, Niigata Cancer Center Niigata Hospital |
| E Shiba | Division of Surgical Oncology, Department of Oncology, Biomedical Research Center Osaka University Medical School |
| H Wakasugi | Department of Internal Medicine, National Kyushu Cancer Center |

### Appendix 2

Judgment Committee

| | |
|---|---|
| Y Sakata | Department of Gastroenterology, Aomori Prefectura Central Hospital |
| S Matsuno | Department of Surgery I, Tohoku University School of Medicine |
| N Moriyama | Department of Radiology, National Cancer Center Hospital East |
| M Yosimori | Department of Internal Medicine, National Cancer Center Hospital |
| T Hayakawa | Department of Internal Medicine II, Nagoya University School of Medicine |

### REFERENCES

Abbruzzese JL, Evans D, Gravel D, Markowitz A, Patt Y and Pazdur R (1995) Docetaxel (D), a potentially active agent for patients with pancreatic adenocarcinomas (PA). *Proc Am Soc Clin Oncol* **14**: 221

Adachi I, Watanabe T, Takashima S, Narabayashi M, Horikoshi N, Aoyama H and Taguchi T (1996) A late phase II study of RP56976 (docetaxel) in patients with advanced or recurrent breast cancer. *Br J Cancer* **73**: 210–216

Bissery MC, Guénard D, Guéritte-Voegelein F and Lavelle F (1991) Experimental antitumor activity of Taxotere (RP56976, NSC628503), a Taxol analogue. *Cancer Res* **51**: 4845–4852

Bissett D and Kaye SB (1993) Taxol and Taxotere: current status and future prospects. *Eur J Cancer* **29A**: 1228–1231

Bruno R, Hille D, Thomas L, Riva A and Sheiner LB (1995) Population pharmacokinetics/pharmacodynamics (PK/PD) of docetaxel (Taxotere) in phase II studies. *Proc Am Soc Clin Oncol* **14**: 457

Cerny T, Kaplan S, Pavlidis N, Schöffski P, Epelbaum R, van Meerbeek J, Wanders J, Franklin HR and Kaye S for the ECTG (Early Clinical Trials Group of the EORTC) (1994) Docetaxel (Taxotere™) is active in non-small-cell lung cancer: a phase II trial of the EORTC early clinical trials group (ECTG). *Br J Cancer* **70**: 384–387

Cortes JE and Pazdur R (1995) Docetaxel. *J Clin Oncol* **13**: 2643–2655

Dieras V, Chevallier B, Kerbrat P, Krakowski I, Roche H, Misset JL, Lentz MA, Azli N, Murawsky M, Riva A, Pouillart P and Fumoleau P (1996) A muticentre phase II study of docetaxel 75 mg $m^{-2}$ as first-line chemotherapy for patients with advanced breast cancer: Report of the clinical screening group of the EORTC. *Br J Cancer* **74**: 650–656

Eisenhauer EA (1995) Docetaxel: current status and future prospects. *J Clin Oncol* **13**: 2865–2868

Fleming TR (1982) One-sample multiple testing procedure for phase II clinical trials. *Biometrics* **38**: 143–151

Francis P, Schneider J, Hann L, Balmaceda C, Barakat R, Phillips M and Hakes T (1994) Phase II trial of docetaxel in patients with platinum-refractory advanced ovarian cancer. *J Clin Oncol* **12**: 2301–2308

Fumoleau P, Chevallier B, Kerbrat P, Dieras V, Le Bail N, Bayssas M and Van Glabbeke M (1993) First line chemotherapy with Taxotere™ (T) in advanced breast cancer (ABC): a phase II study of the EORTC clinical screening group (CSG). *Proc Am Soc Clin Oncol* **12**: 56

Guéritte-Voegelein F, Guénard D, Lavelle F, Le Goff MT, Mangatal L and Potier P (1991) Relationships between the structure of Taxol analogues and their antimitotic activity. *J Med Chem* **34**: 992–998

Japan Society for Cancer Therapy (1993) Criteria for the evaluation of the clinical effects of solid cancer chemotherapy. *J Jpn Soc Cancer Ther* **28**: 101–130

Kunitoh H, Watanabe K, Onoshi T, Furuse K, Niitani H and Taguchi T (1996) Phase II trial of docetaxel in previously untreated advanced non-small-cell lung cancer: A Japanese Cooperative Study. *J Clin Oncol* **14**: 1649–1655

Miller AB, Hoogstraten B, Staquet M and Winkler A (1981) Reporting results of cancer treatment. *Cancer* **47**: 207–214

 © Cancer Research Campaign 1999

Ministry of Health and Welfare (1991) The Japanese guidelines for the clinical evaluation of anti-neoplastic drugs. Mikusu, Tokyo

Rougie P, De Forni M, Adenis A, Ducreux M, Djazouli K, Adams D, Bonneterre J, Clouet P, Blanc C, Bayssas M and Armand JP (1994) Phase II study of Taxotere (RP56976, docetaxel) in pancreatic adenocarcinoma (PAC). *Proc Am Soc Clin Oncol* **13**: 200

Rowinsky EK and Donehower RC (1995) Paclitaxel (Taxol). *N Engl J Med* **332**: 1004–1014

Schiff PB, Fant J and Horwitz SB (1979) Promotion of microtubule assembly in vitro by Taxol. *Nature* **277**: 665–667

Simon R (1989) Optimal two-stage designs for phase II clinical trials. *Controlled Clinical Trials* **10**: 1–10

Taguchi T, Furue H, Niitani H, Ishitani K, Kanamaru R, Hasegawa K, Ariyoshi Y, Noda K, Furuse K, Fukuoka M, Yakushiji M and Kashimura M (1994) Phase I clinical trial of RP56976 (docetaxel) a new anticancer drug (in Japanese, abstract in English). *Jpn J Cancer Chemother* **21**: 1997–2005

Tanigawara Y, Sasaki Y, Otsu T, Fujii H, Kashimura M, Sasaki T, Okumura K and Taguchi T (1996) Population pharmacokinetics of docetaxel in Japanese patients. *Proc Am Soc Clin Oncol* **15**: 479

*© Cancer Research Campaign 1999*
